# Immediate effects of the retrocapital pad and toe bar in sensorimotor foot orthoses on the electromyographic activity of the gastrocnemius muscle in healthy adults

**DOI:** 10.3389/fresc.2026.1887293

**Published:** 2026-09-15

**Authors:** Stephan Becker, Jonas Dully, Alessia Arcieri, Lukas Maurer, Carlo Dindorf, Michael Fröhlich

**Affiliations:** Department of Sports Science, RPTU Kaiserslautern-Landau, Kaiserslautern, Germany

**Keywords:** insoles, orthopedic aids, plantar flexors, proprioception, SMFO

## Abstract

**Background:**

Sensorimotor foot orthoses (SMFOs) regulate muscle tone. Within this growing body of evidence, the impact of two SMFO elements, the retrocapital pad and toe bar, on the plantar flexors has not yet been investigated. It has been observed and postulated that combining these two elements may reduce calf muscle activity.

**Material and methods:**

This randomized crossover study included 29 healthy participants (age, 25.8 ± 6.5 years). Each participant performed 30-seconds walking and running trials on a treadmill to compare the SMFOs and no-foot orthoses (NFOs) conditions. The activities of the gastrocnemius medialis and lateralis were measured using surface electromyography (sEMG). Statistical parametric mapping (SPM) was calculated and analyzed using a paired t-test-like statistic (significance level, *α* = 0.05).

**Results:**

No significant differences in muscle activity were observed between the SMFOs and NFOs conditions during either walking or running in healthy adults.

**Conclusion:**

The effects of SMFOs observed in clinical practice may not appear to extend to healthy young adults.

## 1 Introduction

Foot orthoses are widely used in clinical practice to improve foot, ankle, and knee motion; alleviate pain; correct biomechanical dysfunction; and manage lower-extremity pathologies (1–3). In contrast to conventional foot orthoses, which primarily provide structural support and redistribute plantar pressure, sensorimotor foot orthoses (SMFOs) are designed to additionally modulate neuromuscular activity through targeted stimulation of proprioceptive structures (4–7). This approach has gained increasing attention as a potential strategy for managing various lower-limb pathologies and abnormal movement patterns (6, 8, 9). Achilles tendinopathy, toe walking, and equinus deformity, which are often observed in neurological disorders such as spasticity and Parkinson's disease, are frequently associated with increased tone in the plantar flexors of the upper ankle joint, particularly in the gastrocnemius and soleus muscles (10–12).

The principal mechanism underlying toe walking is persistent overactivity of the gastrocnemius and soleus muscles (13). Ankle-foot orthoses are primarily intended to stabilize the ankle joint, facilitate heel lowering during the stance phase, and promote a normal heel strike. Previous studies have demonstrated that ankle-foot orthoses can reduce muscle activity in the ankle dorsiflexors (14). Lindskov et al. (12) reported that ankle-foot orthoses may reduce spastic activation of the medial gastrocnemius in children with spastic unilateral cerebral palsy; however, they may also functionally suppress tibialis anterior activity during gait. Despite these potential benefits, ankle-foot orthoses can restrict footwear options and may be intrusive during daily activities. In contrast, foot orthoses are considerably less bulky and may therefore offer greater flexibility for everyday use. Within SMFOs, retrocapital pads and toe bars are designed to influence the plantar fascia and toe flexors, thereby indirectly modulating calf muscle activity through fascial and neurophysiological pathways (15). Clinical observations suggest that combining these two elements may improve heel strike; however, this potential effect has not yet been scientifically investigated. Similar observations have been reported for conventional insoles, some of which incorporate comparable elements, although they are not classified as SMFOs (16). This premise is further supported by evidence that increased plantar sensory stimulation may modulate lower-limb muscle activation (17). For example, Jenkings et al. (18) reported normalization of the timing of tibialis anterior activation at initial contact in individuals with Parkinson's disease when walking with a facilitatory ribbed insole compared with a conventional flat insole. Conventional insoles have also been associated with improvements in foot kinematics during toe walking (19). However, findings regarding their effects on calf muscle activity remain inconsistent, and previous studies have not specifically examined the combined use of elements as in this study (20). Therefore, systematic investigation is necessary to warranted to elucidate the mechanisms underlying these clinical observations. Accordingly, this study tested the hypotheses that the muscular activity of the gastrocnemius lateralis and medialis during both walking and running in healthy adults would differ between the SMFOs, incorporating a retrocapital pad and toe bar, and no-foot orthoses (NFO) conditions.

To date, the proposed neuromuscular mechanisms underlying the clinically observed improvement in heel strike have not been empirically investigated. This effect has been hypothesized to be mediated by reduced gastrocnemius muscle activity. Therefore, the present study aimed to investigate whether wearing SMFOs incorporating a retrocapital pad and toe bar alters gastrocnemius activity during gait. Given the lack of previous research in this area, the study first sought to establish whether a general neuromuscular effect could be demonstrated in healthy individuals, who may also use SMFOs for preventive purposes or other complaints.

## 2 Methods

### 2.1 Sample

A total of 38 healthy participants were included in this cross-over study. Nine participants were excluded because of measurement errors, resulting in a final sample of 29 participants (age, 25.8 ± 6.5 years; height, 175.2 ± 9.8 cm; weight, 73.1 ± 12.5 kg; body mass index, 23.6 ± 2.6 kg/m^2^). A sensitivity power analysis was performed for a two-sided paired samples t-test with an alpha level of 0.05. Assuming a statistical power of 80%, the analysis indicated that an effect size of approximately Cohen's *d* = 0.54 could be detected. This analysis was performed to characterize the detectable effect size for the paired design and did not represent a formal power analysis of the one-dimensional statistical parametric mapping (SPM) inference.

All participants were assessed for known or diagnosed foot deformities. Individuals with pronounced foot deformities, diabetes mellitus, or disorders affecting foot sensitivity were excluded. All participants were asymptomatic and able to walk and run without difficulty.

The study was conducted in accordance with the current guidelines of the Declaration of Helsinki and was approved by the responsible ethics committee (No. 99). All participants provided written informed consent after receiving a full explanation of the study procedures and consented to participation and publication of the results.

### 2.2 Procedure

The order of the SMFOs and NFOs conditions was randomized by drawing lots, with each participant completing both conditions in a crossover design. Blinding of participants was not feasible because they could see and feel whether they were wearing the SMFOs. Blinding was therefore limited to the statistical analysis.

Neutral shoes (Adidas Samba; Adidas, Herzogenaurach, Germany) were used to minimize potential effects of additional stabilizing or corrective shoe elements while allowing an individual foot position.

For sEMG measurement of the gastrocnemius, the most prominent points of the lateral and medial heads were identified by palpation while participants stood with their heels raised. Skin preparation and placement of Ag/AgCl adhesive electrodes (Ambu Blue Sensor P, 34-mm diameter, Ambub A/S, Ballerup, Denmark) were subsequently performed in accordance with the SENIAM recommendations (21).

The SMFOs used in this study were specifically manufactured by Springer Aktiv AG (Figure 1) and adapted to the neutral shoes across all available shoe sizes without individual fitting. The SMFOs were composed of ethylene vinyl acetate (EVA) with a Shore hardness of 35. The design incorporated prominent elevations in the retrocapital area (8.7–9.5 mm) and toe bar (7.5–8.1 mm), scaled according to foot size. The medial and lateral elements were ground down to minimize additional influences. The shoes were also equipped with resistive pressure-measurement insoles (T&T Medilogic Medizintechnik GmbH, Germany) to identify heel strike and toe-off for detection of the stance phase (Figure 2). The pressure-measurement insoles were positioned beneath either the SMFO or the original shoe insole, depending on the experimental condition.

**Figure 1:**
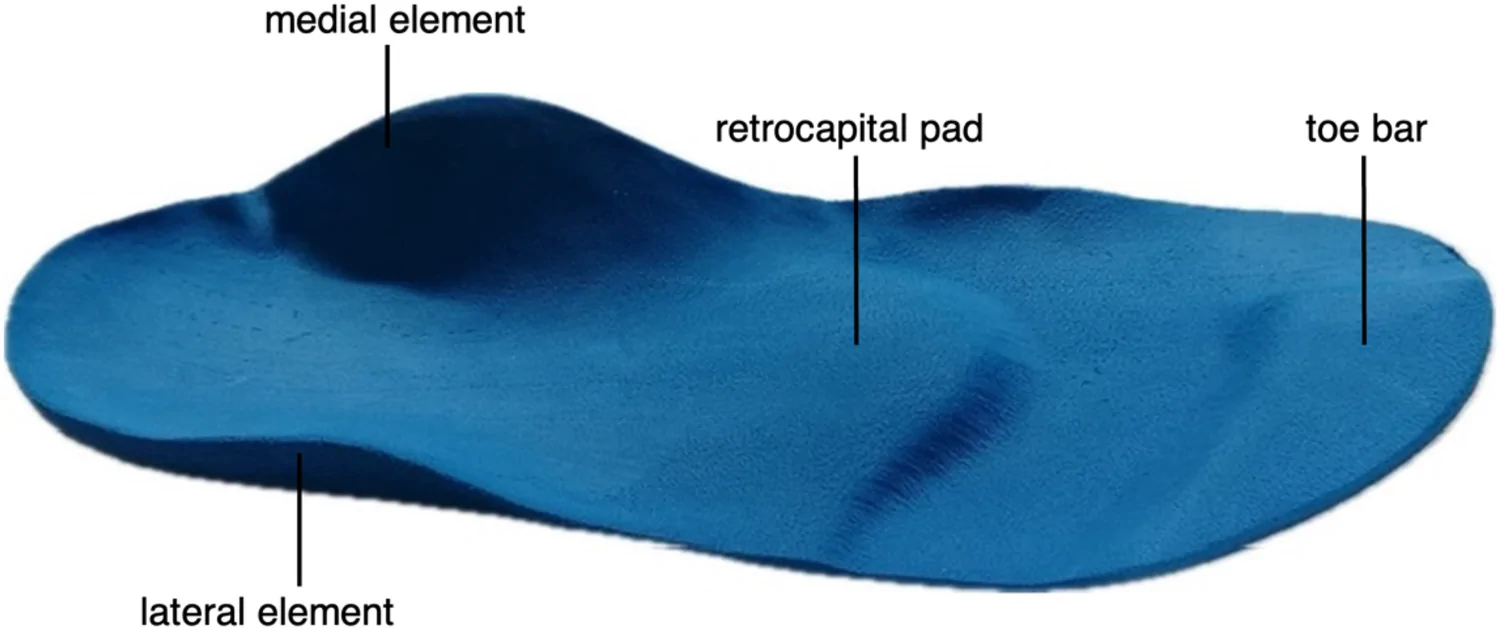
Sensorimotor foot orthoses according to woltring/springer, incorporating a prominent retrocapital pad and toe bar. The medial and lateral elements were ground down to minimize additional influences.

**Figure 2:**
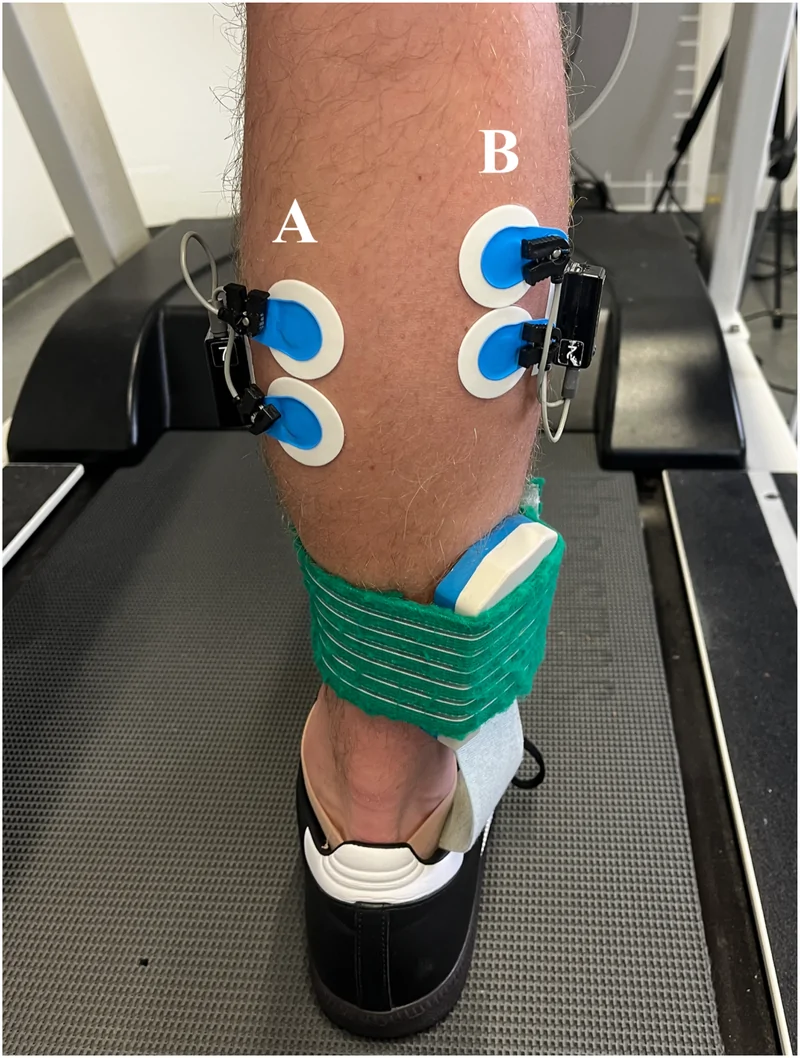
Surface electromyography of **(A)** gastrocnemius medialis and **(B)** lateralis during gait.

Data were collected using telemetric sEMG with the Noraxon Desktop DTS system and corresponding software (MR3 version 3.20.60; Noraxon, Scottsdale, PA, USA) at a sampling frequency 1.500 Hz. Data from the pressure-measurement insoles were integrated into the MR3 software and synchronized with the sEMG data. Participants selected a comfortable treadmill walking speed between 4 and 6 km/h. The selected speed was recorded and maintained across the SMFO and NFO trials. The treadmill protocol consisted of a 5-minute familiarization period followed by a 30-second measurement period, which was comparable to (6), or longer (5) than, the measurement periods reported in previous EMG studies investigating SMFOs. After a 3-minute break, the familiarization period and measurement procedures were repeated under the other condition.

### 2.3 Data processing and analysis

Data were initially processed using Noraxon software (MR3 version 3.20.60, Noraxon, Scottsdale, PA, USA). First, a Bandpass filter (Finite Impulse Response, 20–500 Hz) was applied, and the data were rectified. Subsequently, 20 steps were identified for analysis. The data were processed using MATLAB (version 2024b, The MathWorks, Inc. Natick, Massachusetts, United States), and each step was segmented. Each stance phase, from initial contact to toe-off, was normalized to 1.000 timepoints to enable comparison across steps. SPM was performed separately for medial and lateral gastrocnemius activation across the entire stance phase using a paired t-test, with foot-orthosis condition as the within-participant factor (22). Additionally, the effect size (*d*z) was calculated as Cohen's *d*z, derived directly from the SPM{t} statistic as $dz(t)=\frac{t(t)}{\sqrt{n}}$, which is the standard effect size for paired samples (23). SPM was chosen because it is specifically suited to the analysis of one-dimensional biomechanical and EMG waveform data. The significance level was set at *p* = 0.05. All assumptions required for the statistical analysis were met.

## 3 Results

Descriptive statistics for the integrated EMG (iEMG) activation values during mid-stance (190–500 ‰ of the stance phase) under the SMFOs and NFOs conditions are presented in Table 1.

**Table 1:** Descriptive statistics for time-normalized integrated EMG activation values (20 steps) during walking and running with sensorimotor foot orthoses (SMFOs) and without foot orthoses (NFOs) for gastrocnemius lateralis (GL) and medialis (GM).

Walking						
N = 29	NFO GL	SMFO GL	Δ GL	NFO GM	SMFO GM	Δ GM
Mean	7,032.3	6,990.2	−42.1	9,409.8	8,895.6	−514.2
SD	2,711.9	2,823.0	+111.1	3,079.3	3,123.7	+44.4
Range	8,465.6	9,021.5	+555.9	12,827.1	12,337.6	−489.5
MIN	3,497.0	2,990.5	−506.5	3,187.5	3,611.1	+423.6
MAX	11,962.4	12,012.0	+49.6	16,014.6	15,948.7	−65.9

Running						
N = 29	NFO GL	SMFO GL	Δ GL	NFO GM	SMFO GM	Δ GM
Mean	18,255.6	18,001.8	−253.8	18,516.8	18,003.5	−513.3
SD	3,766.7	3,970.4	+203.7	3,944.6	3,719.4	−225.2
Range	14,798.8	15,709.7	+910.9	15,391.5	20,126.6	+4,735.1
MIN	9,145.3	9,037.9	−107.4	11,646.5	7,498.0	−4,148.5
MAX	23,944.1	24,747.6	+803.5	27,038.1	27,624.6	+586.5

SD, standard deviation; MIN, minimum; MAX, maximum; Δ, difference between GL and GM.

The mean normalized sEMG profiles and corresponding standard deviations for all participants under the SMFO and NFO conditions are shown in Figures 3A–6A, with Figures 3A, 5A presenting the gastrocnemius lateralis and Figures 4A, 6A presenting the gastrocnemius medialis. The two conditions exhibited highly similar activation profiles for both muscles during walking and running throughout the stance phase, with largely overlapping standard deviations. In the corresponding SPM curves (Figures 3B–6B), the test statistic (black line) remained within the critical threshold lines (dashed red lines) throughout the stance phase. Accordingly, no statistically significant differences were detected between the SMFO and NFO conditions in the gastrocnemius lateralis and medialis activity during either walking or running. Therefore, neither Hypothesis 1 nor 2 was supported.

**Figure 3:**
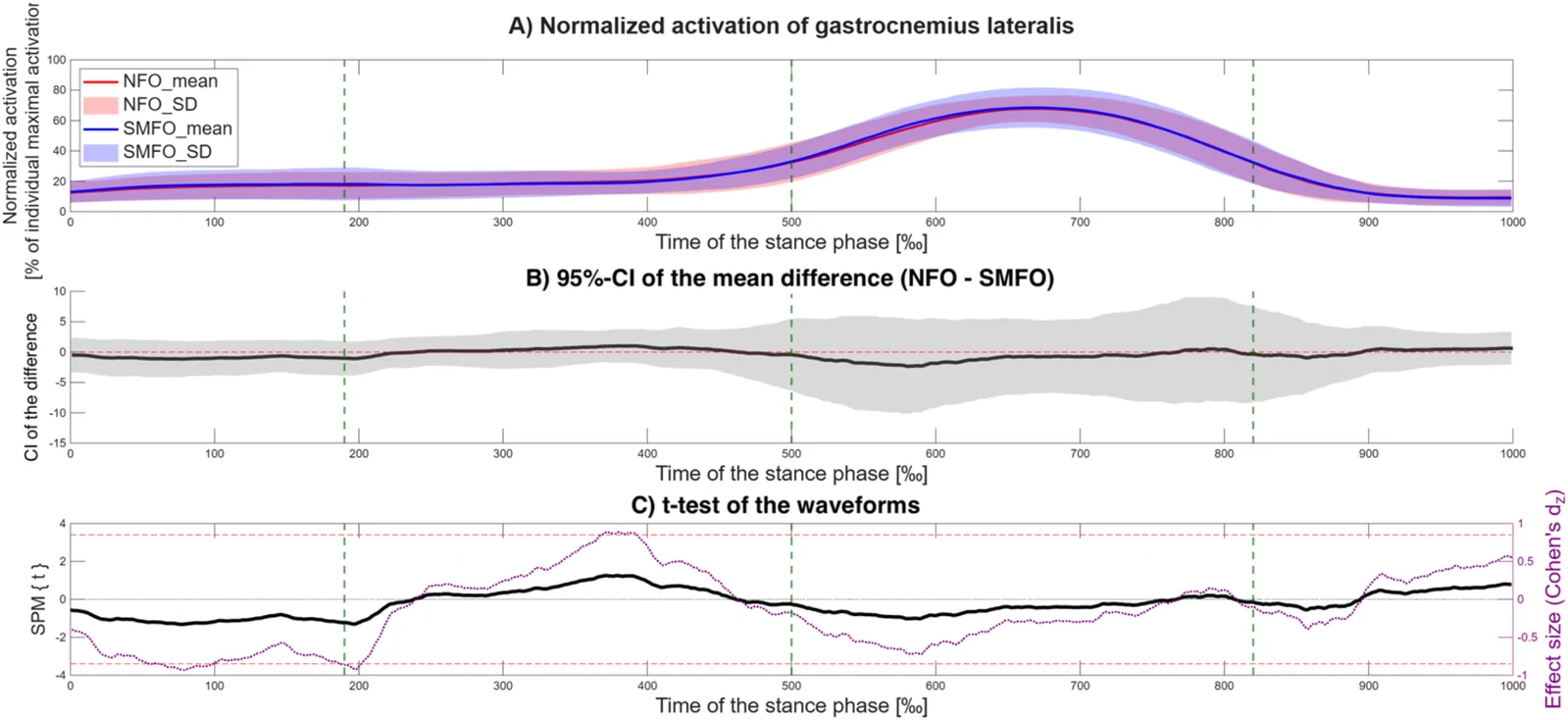
Walking**. (A)** Normalized activation of gastrocnemius lateralis during the stance phase of walking. Vertical green dotted lines separate the stance phases. **(B)** 95% confidence interval for the mean difference (NFO−SMFO). **(C)** *t*-test of the waveforms during walking (bold black line, *t*-value). Statistical significance is present when the statistical parametric mapping (SPM) value exceeds the threshold (horizontal red dashed lines). The purple dotted line represents the effect size. Vertical green dotted lines separate the stance phases.

**Figure 4:**
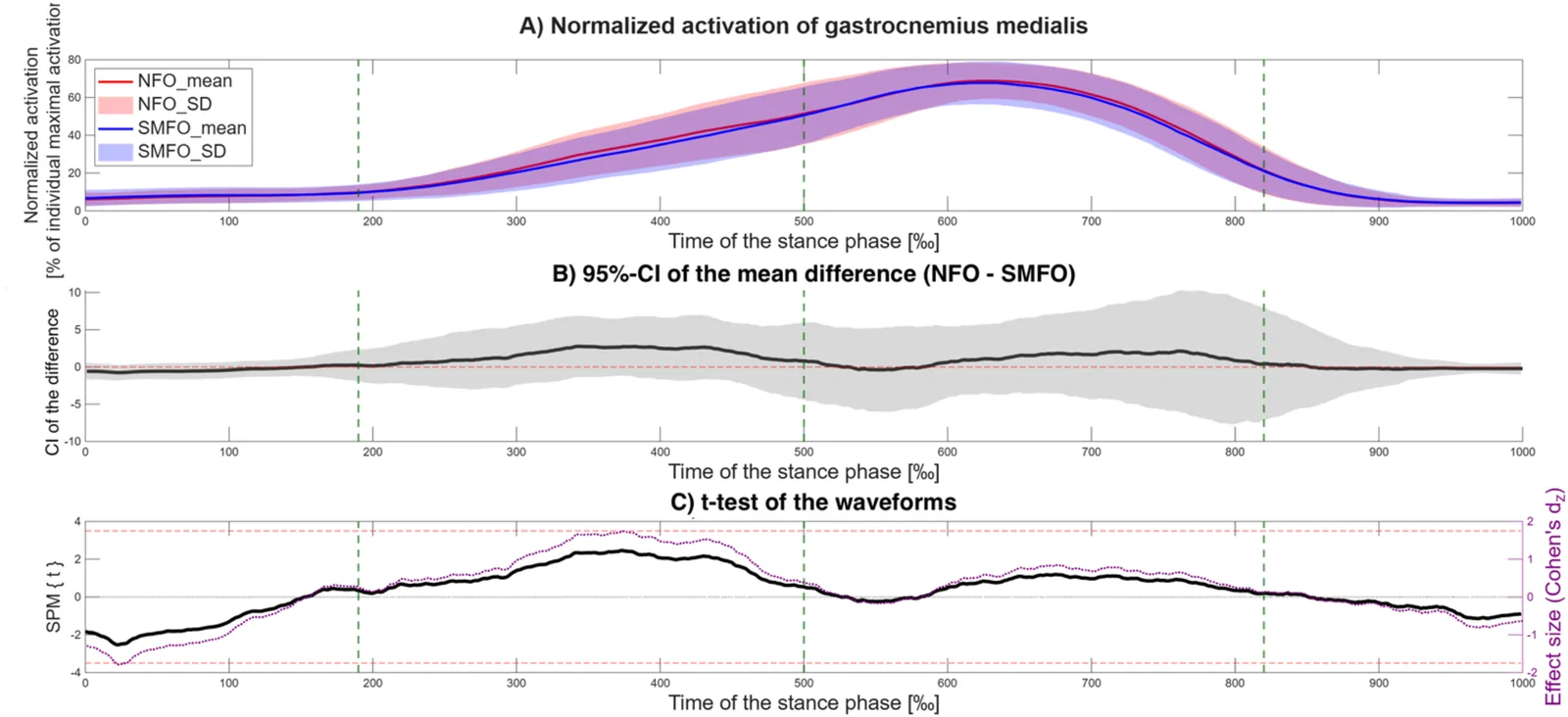
Walking**. (A)** Normalized activation of gastrocnemius medialis during the stance phase of walking. Vertical green dotted lines separate the stance phases. **(B)** 95% confidence interval for the mean difference (NFO−SMFO). **(C)** *t*-test of the waveforms during walking (bold black line, *t*-value). Statistical significance is present when the statistical parametric mapping (SPM) value exceeds the threshold (horizontal red dashed lines). The purple dotted line represents the effect size. Vertical green dotted lines separate the stance phases.

**Figure 5:**
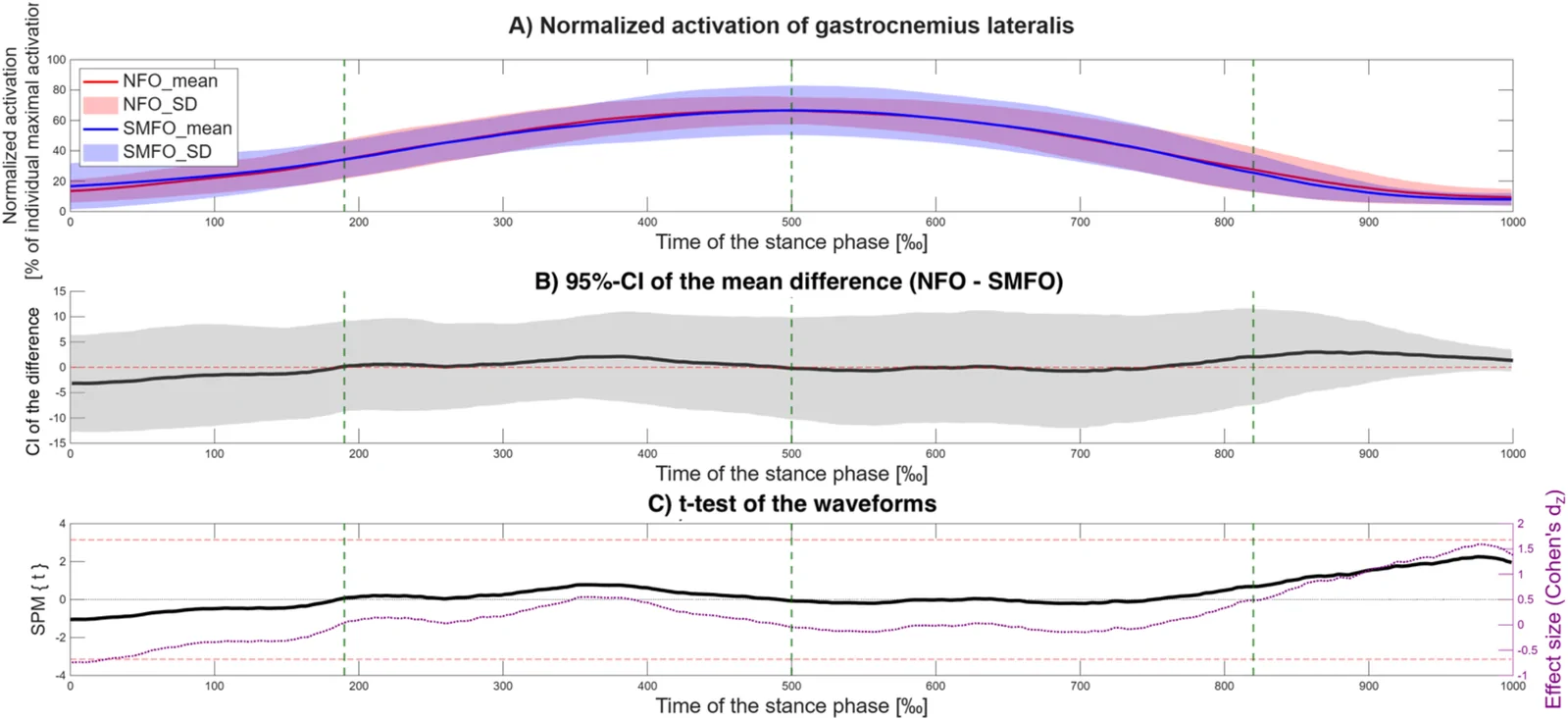
Running**. (A)** Normalized activation of gastrocnemius lateralis during the stance phase of running. Vertical green dotted lines separate the stance phases. **(B)** 95% confidence interval for the mean difference (NFO−SMFO). **(C)** *t*-test of the waveforms during running (bold black line, *t*-value). Statistical significance is present when the statistical parametric mapping (SPM) value exceeds the threshold (horizontal red dashed lines). The purple dotted line represents the effect size. Vertical green dotted lines separate the stance phases.

**Figure 6:**
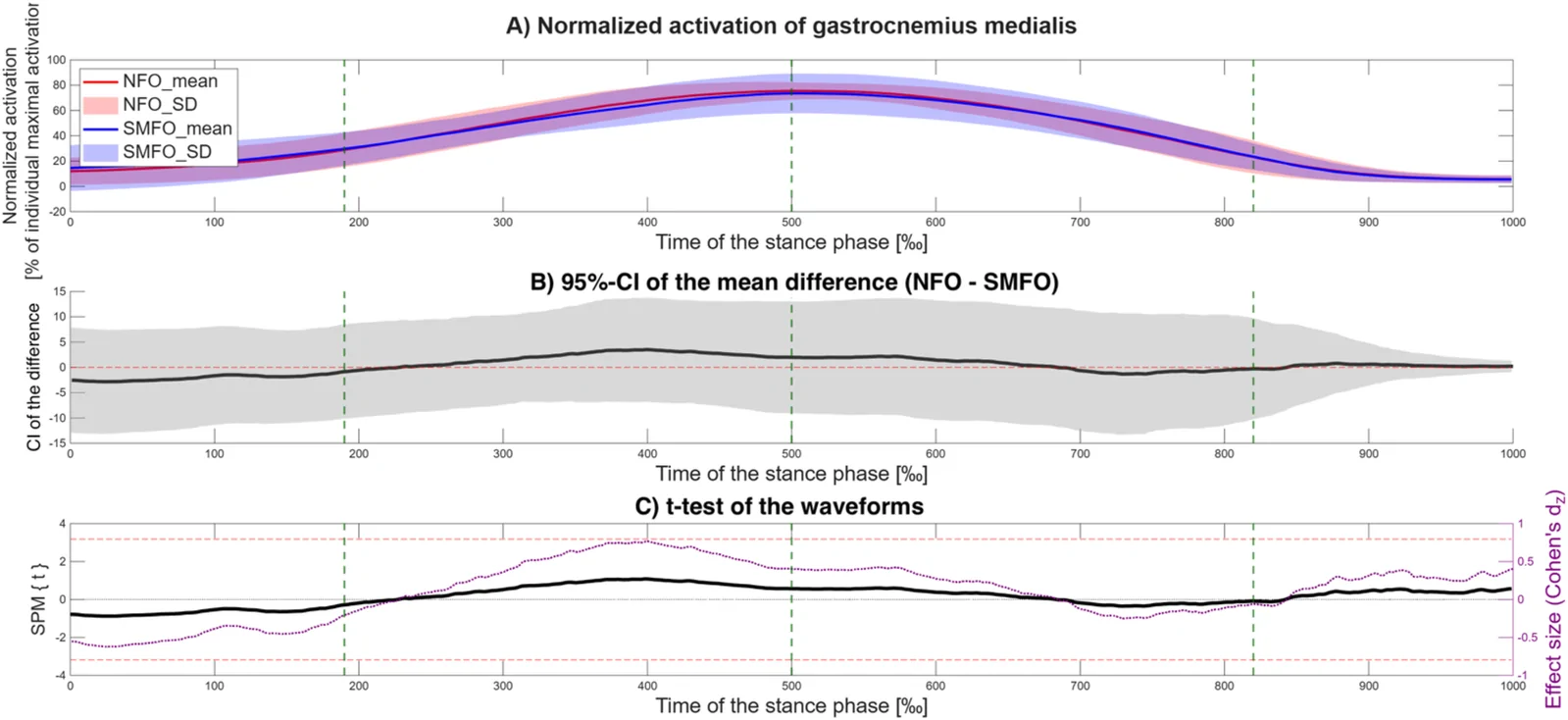
Running**. (A)** Normalized activation of gastrocnemius medialis during the stance phase of running. Vertical green dotted lines separate the stance phases. **(B)** 95% confidence interval for the mean difference (NFO−SMFO). **(C)** *t*-test of the waveforms during running (bold black line, *t*-value). Statistical significance is present when the statistical parametric mapping (SPM) value exceeds the threshold (horizontal red dashed lines). The purple dotted line represents the effect size. Vertical green dotted lines separate the stance phases.

## 4 Discussion

This study investigated the immediate effects of SMFO on gastrocnemius sEMG activity in healthy adults. The positive clinical findings previously attributed to modulation of gastrocnemius activity were not confirmed in the present study (4). No significant immediate differences in medial and lateral gastrocnemius activity were observed between the SMFO and NFO conditions during either walking or running in healthy participants. Therefore, neither of the hypotheses were supported.

The retrocapital pad straightens the transverse arch of the foot and redistributes plantar pressure (4, 16). These effects may be relevant to various symptoms, including metatarsal pain (16), which is often associated with a lowered transverse arch (splayfoot) (24). In contrast, the toe bar is intended to counteract the development of hammer and claw toes (25, 26) and may improve toe flexor strength (26) and balance (27).

The combination of a toe bar and retrocapital element is also used in orthopedic practice for lower-extremity conditions associated with increased calf muscle tone, such as toe walking, Achilles tendon pain, and neurological disorders (4). From a mechanical perspective, it has been hypothesized that combining these elements increases tension in the plantar fascia and toe flexors (28). Unlike a flat surface, such as that provided by a conventional shoe or the floor, this combination creates a concave region into which the metatarsal head can sink. This configuration could elongate the plantar structures and lead to increased tension (28), resembling the windlass mechanism (29), which could, in turn, result in retroversion of the calcaneus (30). The windlass mechanism is typically initiated during the terminal stance phase as the plantar fascia is stretched by the rolling motion of the foot and toe extension (29, 30). This process is normally supported by the calf muscles, which exhibit substantial activity between terminal stance and push-off to propel the body forwards and rotate the calcaneus into an inverted position (31). Although complex and currently unsupported by empirical evidence, this proposed mechanism could potentially explain the beneficial effects observed in clinical practice.

An alternative and potentially more plausible neuromuscular response to this proposed stretching mechanism, albeit one that contradicts the clinical observations, would be increased gastrocnemius activity. Retroversion of the calcaneus and associated transmission of tension to the calf through the fascial system could stimulate Ia afferents from muscle spindles in the gastrocnemius (31, 32). The resulting stretch reflex would be expected to increase gastrocnemius activation to resist further muscle lengthening and tension (31, 32).

Another possible explanation is an avoidance response to the mechanical stimuli produced by the SMFO profile (16, 20). Although this mechanism would be less specific, it could nevertheless be beneficial in certain pathological conditions by reducing forefoot loading duration and promoting heel strike (19). The SMFO profile may provide plantar sensory input that induces an adaptation in the gait pattern, with the foot transitioning more rapidly from forefoot loading towards heel contact. In individuals with pathological forefoot gait, such an adaptation could promote a more physiological gait pattern (19, 33). Consequently, calf muscle iEMG from initial contact to mid-stance could also decrease because less time is spent loading the forefoot.

To date, comparable research has primarily investigated conventional insoles, also referred to as biomechanical foot orthoses (15). Several studies have examined the effects of conventional foot orthoses on gastrocnemius sEMG activity without incorporating forefoot elements such as a retrocapital pad and toe bar (1, 34–39). These studies have predominantly investigated medial and lateral wedges, often in individuals with conditions such as pes planus or pes planovalgus, and their effects on lower-limb muscle activity. Some studies have reported reduced gastrocnemius activity (35, 36), potentially because of the mechanical support provided to overpronated or everted feet. However, the SMFOs used in the present study intentionally incorporated no medial or lateral elements; therefore, direct comparison with these findings is limited. Vermand et al. (16) examined the effect of a retrocapital pad on posture and static alignment in 48 healthy runners and reported significant posterior displacement of the center of pressure, hips, shoulders, and ears. Although electromyographic outcomes were not analyzed, this posterior displacement could potentially reduce tension in the plantar flexors. Regarding the toe bar, Nakano et al. (40) examined its influence on the sEMG activity of several lower-extremity muscles in 12 participants and reported significantly increased gastrocnemius activity. In the context of static balance, another study investigated the same combination of forefoot elements and demonstrated improved static balance with SMFOs compared with NFOs in healthy adults (28). To the best of our knowledge, however, no previous study has examined the combined effects of these two elements on gastrocnemius activity in either conventional foot orthoses or SMFOs.

Although the available evidence indicates that SMFOs may have beneficial effects on kinematic (41–44), kinetic (41), and electromyographic outcomes (5, 6, 43, 45, 46), the physiological mechanisms underlying these effects remain largely theoretical. Several studies (5, 6, 43, 45, 46) have demonstrated significant changes in sEMG activity following the use of SMFOs, particularly in the fibular muscles (5, 6), which may contribute to improvements in various symptoms. However, the neurophysiological mechanisms responsible for these effects remain unclear.

From a clinical perspective, the present findings do not support the use of SMFOs incorporating a retrocapital pad and toe bar specifically to reduce gastrocnemius activity in healthy young adults with normal gait patterns. Importantly, these findings should not be extrapolated to patient populations. Rather, the absence of an effect in healthy individuals suggests that the proposed neuromuscular response may depend on the presence of an underlying gait abnormality or neuromuscular impairment. Accordingly, the present findings do not support recommending SMFOs solely for the purpose of reducing gastrocnemius activity in healthy individuals. Their clinical use should instead be guided by individual functional impairments and therapeutic goals (39, 47).

A major limitation of the present study was the inclusion of young, healthy participants with normal gait patterns. Such individuals may have limited potential for orthosis-induced changes in gait or muscle activity compared with individuals with pathological forefoot gait, increased plantar flexor tone, or other neuromuscular dysfunctions (39). In individuals with toe walking or pronounced forefoot loading, deviations from a physiological gait pattern may be greater, and changes in muscle activity in response to orthotic intervention may therefore be more readily detectable. Furthermore, underlying pathologies may alter sensorimotor function and thereby influence responses to orthotic interventions (48, 49). Another limitation was that the SMFOs were manufactured for each shoe size by an experienced master craftsman rather than individually fitted to each participant. Consequently, the retrocapital pads and toe bars were based on an average configuration relative to shoe size rather than individual foot morphology. This lack of individualization may have reduced the intended sensorimotor stimulus.

To the best of our knowledge, this is the first study to investigate the effects of SMFOs incorporating a retrocapital pad and toe bar on gastrocnemius muscle activity. Future studies should investigate clinically relevant populations, particularly individuals in whom increased plantar flexor activity or altered gait patterns form part of the clinical presentation. Longitudinal studies are also warranted to determine the potential medium- and long-term effects of SMFOs on muscle activity, kinematics, and kinetics.

## 5 Conclusion

The present findings do not provide evidence that SMFOs incorporating a toe bar and retrocapital pad alter gastrocnemius muscle activity in young adults with normal gait patterns. No significant differences between the SMFO and NFO conditions were observed during either walking or running across the stance phase. Further studies in larger clinical populations are needed to confirm and validate these findings.

## Data Availability

The raw data supporting the conclusions of this article will be made available by the authors, without undue reservation.
